# Forest type and height are important in shaping the altitudinal change of radial growth response to climate change

**DOI:** 10.1038/s41598-018-37823-w

**Published:** 2019-02-04

**Authors:** Penghong Liang, Xiangping Wang, Han Sun, Yanwen Fan, Yulian Wu, Xin Lin, Jinfeng Chang

**Affiliations:** 1 College of Forestry, Beijing Forestry University, Beijing, 100083 China; 2 Department of Ecology, College of Environmental Sciences, and Key Laboratory for Earth Surface Processes of the Ministry of Education, Peking University, Beijing, 100871 China

## Abstract

Tree radial growth is widely found to respond differently to climate change across altitudinal gradients, but the relative roles of biotic factors (e.g. forest type, height and density) vs. climate gradient remain unclear. We sampled tree rings from 15 plots along a large altitudinal gradient in northeast China, and examined how climate gradient, forest type, height, tree size and density affect: (1) temporal growth variability [mean sensitivity (MS) and standard deviation (SD) of the chronologies], and (2) the relationship of ring width indices (RWI) with historical climate. We used BIC based model selection and variable importance to explore the major drivers of their altitudinal patterns. The results showed that: both growth variability and RWI-climate relationships changed significantly with altitude. Forest height was the most important predictor for altitudinal changes of MS and SD. For RWI-climate relationships, forest type was more important than climate gradient, while height and stem density were weak but necessary predictors. We showed that the altitudinal difference in growth response to climate change cannot be explained by climate gradient alone, and highlight the necessity to examine the influence of biotic factors (which covary with climate across geographic gradient) to better understand forest response to climate change.

## Introduction

The world is undergoing rapid climate change^1^, and forest growth has been widely reported to respond to climate change differently across climatic gradients^2–4^. Understanding the drivers of these different responses is critical for predicting forest dynamics under future climate and to develop adaptive strategies^4–6^, especially when climate change has reduced tree productivity and survival in many regions of the world^7,8^.

Altitudinal gradients are ideal places to examine the different response of tree growth to climate change across environmental gradients. Many studies have found that the relationship between ring width indices (RWI) and climate varies with altitude (e.g. ref.^9–11^) and latitude^2,12,13^. However, many studies have included only one forest zone or examined the different growth response of a single species across elevations (e.g. ref.^10,14–16^). Consequently, the altitudinal change of RWI-climate relationship was generally explained as a result of different climate at different elevations. However, large altitudinal gradients generally cover different forest zones. This is similar to the latitudinal gradient and thus more useful in understanding the different growth response to climate change at broad scales. Other studies have found that different forest types (species) under a similar climate may show contrasting growth response to climate change, suggesting that species identity is another key factor leading to different growth response^4,11,17,18^. Thus, different forest types distributed along elevational gradients may contribute to the altitudinal change of RWI-climate relationship, but its relative effect vs. climate gradient has not been well quantified.

In addition to forest type, other biotic factors may also influence the growth response to climate change. For example, greater tree height leads to greater difficulty in transporting water from root to canopy^19,20^, thus the growth of taller trees may be more sensitive to water deficit than small trees under the same climate condition. Consistent with this prediction, some studies have found that the growth of larger-DBH or older trees (which were generally taller trees) were more sensitive to climate change^6,21,22^. Meanwhile, tree density was also found to have significant influence on growth sensitivity to water deficit (e.g. ref. ^6^). Tree height and stem density are widely observed to change markedly with altitude and latitude^23–26^. Thus the changes of RWI-climate relationship may not be the sole result of climate gradient and different forest types across latitude (altitude), tree height, size and density may also play a significant role. Here we tested this hypothesis along a large altitudinal gradient.

While most studies have focused on the altitudinal pattern of the growth-climate relationship, another important aspect of growth response to climate change, i.e. temporal growth variability, has remained less examined. Studies have found that chronology statistics, e.g. mean sensitivity (MS) and standard deviation (SD), also changed across altitudes^14,16,27^. MS is a measure of year-to-year growth variability, and is commonly accepted to reflect the growth sensitivity to high-frequency variation in climate. Meanwhile, SD reflects the multi-decadal growth variability in a chronology, which was related to the low-frequency fluctuation in climate^28,29^. Thus, the altitudinal changes of MS and SD can provide valuable information on how growth response to climate change varies along altitudinal gradients. However, the two metrics have largely been used as indicators of chronology quality in many studies, and the abiotic and biotic drivers for their geographic patterns have been less explored^30,31^. Consequently, a second aim of our study is to examine this question. Meanwhile, RWI-climate relationship and growth variability are distinctive aspects of growth response to climate change. Thus the relative roles of climate, forest type, height and stem density on their altitudinal patterns are expected to be different. A third aim of this analysis is to examine how the abiotic and biotic drivers differ between growth variability and RWI-climate relationship.

We sampled tree rings from 15 plots along the altitudinal gradient of Mt. Changbai to examine the drivers of different growth response to climate change. Specifically, we asked three questions as follows. (1) How does RWI-climate relationship and growth variability change across different forest types along the altitudinal gradient? (2) Does biotic factors (including forest type, height, size and density) affect the altitudinal change of RWI-climate relationship and growth variability? (3) What are the differences in the abiotic and biotic drivers between RWI-climate relationship and growth variability?

## Materials and Methods

### The study area

Mt. Changbai (41°43′–42°26′N, 127°42′–128°17′E) is situated at the southeast border of Jilin Province, northeast China. The climate in this region is characterized by warm summers, cold winters, abundant precipitation and a short growing season, largely controlled by the East Asian monsoon. With increasing altitude from mountain foot to top, mean annual temperature decreases from 4.9 to −7.3 °C and mean annual precipitation increases from 600 to 1340 mm^32^. As a result, different forest zones are distributed along the altitudinal gradient of Mt. Changbai. (1) 500–1100 m: Korean pine and broadleaf mixed forest (KBF), dominated by *Pinus koraiensis* and mixed with broadleaf species, such as *Quercus mongolica*, *Tilia* spp. In this forest zone there are also secondary forests of KBF, e.g. *Betula platyphylla* forests (BPF). (2) 1100–1700 m: evergreen needle-leaved forest (ENF), composed of *Picea jezoensis* and *Abies nephrolepis*; and deciduous needle-leaved forest (DNF) dominated by *Larix olgensis*. (3) 1700–2000 m: *Betula ermanii* forest (BEF)^33^. Thus, the mountain provided an idea place to examine the abiotic and biotic drivers for altitudinal change of growth response to climate change.

### Field sampling and chronology development

To examine the altitudinal change of tree growth response to climate change, we established 15 plots (20 * 50 m) from 750 m to 1990 m (Table 1). These plots covered all the major forest types distributed along the altitudinal gradient on the north slope of Mt. Changbai, including timberline forests (BEF, three plots), subalpine forests (four ENF plots, and two DNF plots), temperate forests (four KBF plots and two BPF plots). In each plot, we documented geographic coordinates (latitude, longitude and altitude). For each tree with a diameter at breast height (DBH) > 3 cm, the DBH and tree height were measured. In each plot we selected 25 canopy trees of one or two dominant species (see Table 1 for the species sampled), and extracted two tree-ring cores at 1.3 m from two vertical directions. The cores were sampled in the summer of 2006.

**Table 1:** Site information and summary statistics for the residual chronologies of the plots along the altitudinal gradient of Mt. Changbai.

Plot	Altitude (m)	Forest type	Species sampled	Chronology length (year)	No. of cores	MS	SD	RBAR	SNR	EPS
CBS1	1985	BEF	*Betula ermanii*	1914–2005	25	0.404	0.293	0.434	12.279	0.925
CBS2	1910	BEF	*Betula ermanii*	1828–2005	26	0.336	0.297	0.531	27.131	0.964
CBS3	1884	BEF	*Betula ermanii*	1850–2005	24	0.314	0.289	0.463	15.532	0.940
CBS4	1733	ENF	*Picea jezoensis, Abies nephrolepis*	1874–2005	29	0.236	0.202	0.359	15.684	0.940
CBS5	1674	ENF	*Picea jezoensis, Abies nephrolepis*	1825–2005	28	0.315	0.265	0.421	8.008	0.889
CBS6	1536	ENF	*Picea jezoensis, Abies nephrolepis*	1818–2005	24	0.232	0.219	0.263	5.711	0.851
CBS7	1440	DNF	*Larix olgensis*	1952–2005	28	0.235	0.209	0.397	7.242	0.879
CBS8	1436	DNF	*Larix olgensis*	1807–2005	32	0.27	0.227	0.457	11.801	0.922
CBS9	1370	ENF	*Picea jezoensis, Abies nephrolepis*	1838–2005	30	0.308	0.28	0.416	9.992	0.909
CBS10	1114	KBF	*Pinus koraiensis*	1903–2005	28	0.198	0.182	0.21	3.726	0.788
CBS11	1054	BPF	*Betula platyphylla*	1946–2005	30	0.176	0.166	0.321	8.044	0.889
CBS12	1034	KBF	*Pinus koraiensis*	1847–2005	23	0.223	0.206	0.232	2.713	0.731
CBS13	930	KBF	*Pinus koraiensis*	1855–2005	26	0.203	0.175	0.334	6.024	0.858
CBS14	763	KBF	*Pinus koraiensis*	1911–2005	24	0.189	0.171	0.293	4.982	0.833
CBS15	750	BPF	*Betula platyphylla*	1942–2005	32	0.288	0.274	0.331	5.929	0.856

Abbreviations: MS, mean sensitivity; SD, standard deviation; RBAR, mean correlation among all tree-ring series; SNR, signal-to-noise ratio; EPS, expressed population signal; BEF, *Betula ermanii* forest; ENF, evergreen needle-leaved forest; BPF, *Betula platyphylla* forest; KBF, mixed broad- and needle-leaved forest; DNF, deciduous needle-leaved forest.

All cores were mounted and sanded with successively finer grades of sandpaper until annual rings could be easily distinguished. Tree-rings were cross-dated visually using the skeleton plot method^34^, and the ring widths were measured with an accuracy of 0.01 mm using the LINTAB6 measurement system and TSAP software (Frank Rinn Co. Ltd., Germany). The quality of cross-dating and measurement was checked by cross-correlation analysis using the software COFECHA^35^. Some cores were excluded because they could not be well measured or cross-dated. We then constructed a chronology for each plot, using the remaining 23–32 cores (Table 1). The growth trends (decreasing ring-width with increased age) were removed from raw ring-width data with exponential curves, which was necessary for examining the high-frequency growth variation associated with climate changes^28^. A residual chronology that maximized the climatic signal was then created for each forest type using the ARSTAN software^36^. As shown in Table 1, the majority chronologies had an expressed population signal (EPS) >0.8 except in two cases (which were not far from 0.8). This suggests that the qualities of the chronologies were good enough for further study^37^.

### Climate data

Monthly climate data between 1959 and 2005 of the Erdao meteorological station, at the foot of Mt. Changbai (591 m), were obtained from the China National Climatic Data Center (http://data.cma.cn/). Our plots differed greatly in altitude and thus, for a better analysis of the growth-climate relationship, we estimated the historical monthly climate for each plot. For altitudinal changing rates of monthly mean temperature and total precipitation (see Supplementary Table S1), we used the model of Wang *et al*.^38^. The model was developed based on 48 climate stations in the Mt. Changbai region (including the Tainchi station at the mountain top, 2623 m), and has been calibrated by independent climate data which showed that the model was accurate enough (the *R*^2^ between the estimated and measured values was 0.83 for temperature and 0.79 for precipitation, respectively)^38^. For each plot, the climate variables in each month by each year (1959 to 2005) were estimated based on the altitudinal changing rates of temperature and precipitation in Supplementary Table S1. Then the RWI of the plot was related to these monthly climate variables to examine the growth response to climate change (see Supplementary Table S2).

We also calculated the multi-decades means of monthly temperature and precipitation for each plot, which were then used to calculate climate indices such as actual evapotranspiration (AET), mean annual temperature and precipitation, mean temperature for the coldest month, etc. These multi-decades mean climate indices were used to depict the climate condition of each plot, in order to evaluate the relative effect of climate gradient vs. biotic factors (e.g. forest type and height) on altitudinal patterns of growth response to climate change.

### Statistical analyses

We used MS and SD of the chronologies as indicators of temporal growth variability of radial growth. For growth-climate relationship, we related RWI of each plot to monthly temperature and precipitation from June of the previous year through September of the current year (Supplementary Table S2). Because we are interested in the abiotic and biotic drivers for the altitudinal change of RWI-climate relationship, we selected some correlations for further statistical analyses based on two criterions: (1) the correlation should show significant change with altitude (*P* < 0.05); (2) the monthly climate variable should have significant correlation (*P* < 0.05) with RWI in at least three plots. As a result, the following correlations were selected: the correlation of RWI with temperature and precipitation of current June and previous July (Cor_c6T, Cor_c6P, Cor_p7T and Cor_p7P, respectively), and the RWI correlation with current July temperature (Cor_c7T) and previous June precipitation (Cor_p6P). We also conducted a principal component analysis (PCA) to check the results (Supplementary Fig. S1), using the correlations between RWI and monthly climate variables of the 15 chronologies. The PCA1 loadings of the 15 plots were highly correlated with elevation (*r* = 0.84, *P* < 0.001), suggesting that PCA1 can well reflect the altitudinal patterns of RWI-climate correlations. As shown in Supplementary Fig. S1, the PCA1 scores (absolute value) were the highest for Cor_p7T, Cor_c6T, and Cor_c7T (Supplementary Fig. S1b), and Cor_p6P, Cor_p7P and Cor_c6P (Supplementary Fig. S1c). Thus the PCA results confirmed that the correlations we selected were major components of altitudinal change of growth response to climate change, and are well suited for our purpose. While there were still some other significant correlations in Supplementary Table S2, they did not reveal clear altitudinal patterns or occurred in only one or two plots. These correlations may be caused by local abiotic and biotic factors specific to the plots, which were not our focus and not further analyzed.

To examine the potential drivers for altitudinal changes of growth variability (MS, SD) and RWI-climate correlations, we used general linear models (GLM) to explain these variables with explanatory terms as follows. (1) Altitudinal climate gradient. We used AET to depict the climate condition of the plots, because AET reflects the simultaneous availability of energy and water and is widely used as proxy of climate productivity of vegetation^26,39^. Other climate variables, such as mean annual temperature and precipitation, were highly correlated with AET and thus not used to avoid collinearity. (2) The forest type of each plot (see Table 1). (3) Forest height. Here we used the maximum tree height of the plot, which is a commonly used surrogate (e.g. ref.^40,41^). We also used the mean height for the trees that tree-rings were sampled to repeat the statistical analyses, and the results were similar as using forest height. This is not surprising because we cored dominant canopy trees. (4) Stem density of each plot. (5) Maximum stock volume for the trees in a plot. Previous studies have found that RWI-climate relationship differed between large- and small-DBH trees^6,21^, suggesting the potential role of tree size on growth response to climate change. We used volume instead of DBH because volume is a better metric for tree size than DBH or height (e.g. ref.^42^). In the preliminary statistical analyses, we have included interactions in GLMs. However, the interaction terms were not significant or showed only weak effects, and thus were excluded from the final analyses.

To identify the most important factors affecting growth variability and RWI-climate relationships, we first used the model selection approach based on Bayesian Information Criterion (BIC) to obtain the most parsimonious model, which is more conservative in retaining variables than commonly used Akaike Information Criterion (AIC)^43^. In a next step, we evaluated the relative importance of each variable retained in the final model, using the R package of “relaimpo 2.2–2”^44^. The package adopts the “LMG” approach to yield natural decompositions of model *R*^2^ in regression models^45^. This approach clearly identifies a variable’s contribution by itself and in combination with all other predictors^46^, and thus, has been not only recommended by statisticians because it is superior to other importance metrics, but also increasingly used in ecological studies^44,46,47^.

## Results

### Altitudinal patterns of growth variability and growth-climate relationship

**Figure 1:**
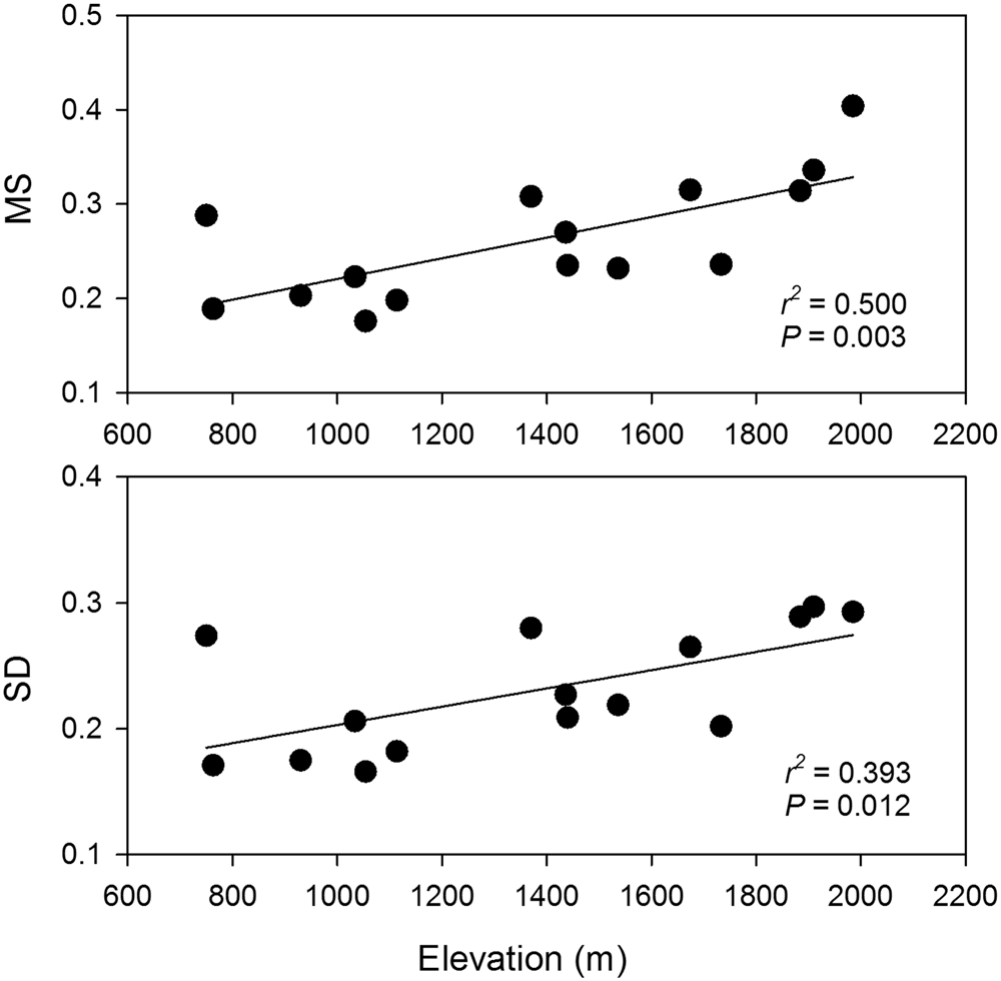
Changes of two chronology statistics, i.e. mean sensitivity (MS) and standard deviation (SD), for 15 plots along the altitudinal gradient of Mt. Changbai.

The statistics for chronology of each plot were reported in Table 1. For the two indicators of temporal growth variability, MS ranged between 0.176 and 0.404, and SD ranged between 0.166 and 0.297. Both MS and SD increased significantly with higher altitude, with an *R*^2^ of 0.50 and 0.39, respectively (Fig. 1).

**Figure 2:**
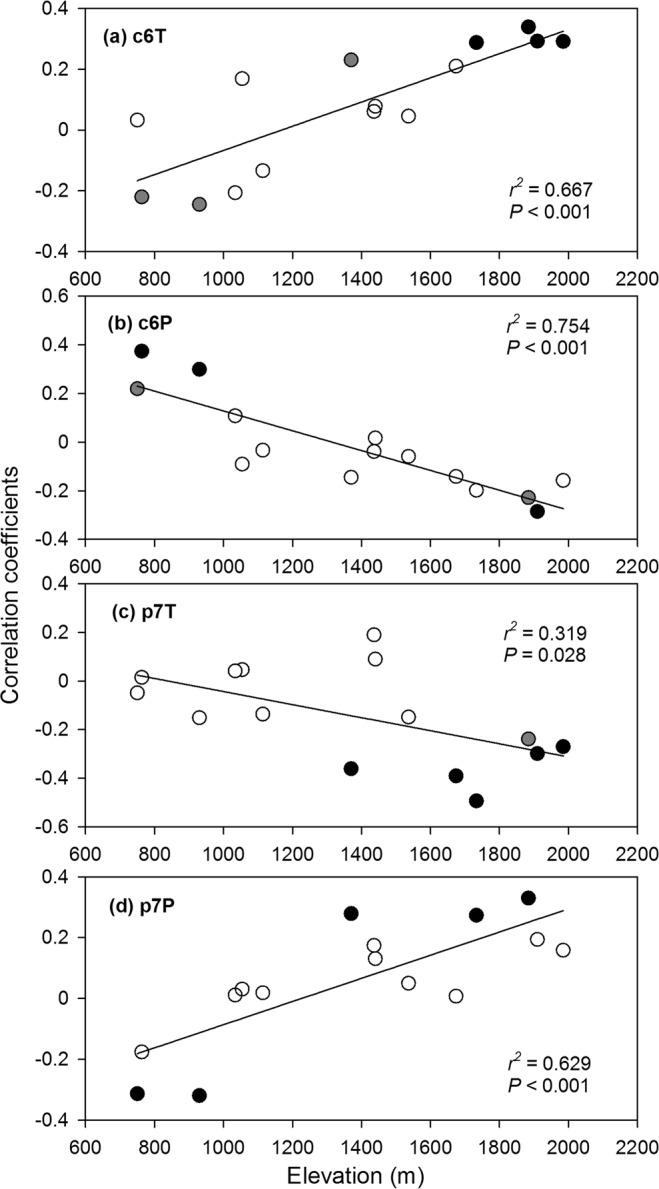
Changes of correlations between ring-width indices and some monthly climate variables along altitudinal gradient. (**a**) Temperature of the current June (c6T). (**b**) Precipitation of the current June (c6P). (**c**) Temperature of the previous July (p7T). (**d**) Precipitation of the previous July (p7P). Black dotes denote RWI-climate correlations with *p* values < 0.05; grey dots, *p* < 0.1; Circles, *p* > = 0.1.

The correlations of RWI with monthly climate variables were reported for each plot in Supplementary Table S1. There were six correlations that showed significant altitudinal patterns (i.e. met the two criterions that we set in the Methods). The correlation of RWI with current June (Cor_c6T) changed from negative at low elevations to positive at high altitudes (Fig. 2a). The altitudinal pattern of Cor_c7T was similar and thus not drawn (*R*^2^ = 0.65). Conversely, the correlation of RWI with current June precipitation (Cor_c6P) changed from positive at low altitudes to negative at high altitudes (Fig. 2b). Climate in the previous summer also showed clear altitudinal patterns. RWI’s correlation with previous July temperature (Cor_p7T) was negatively related to altitude (Fig. 2c), while the correlations with previous July (Cor_p7P, Fig. 2d) and June precipitation (Cor_p6P, *R*^2^ = 0.43, similar as Cor_p7P and thus not shown) were positively related to altitude.

### Drivers for altitudinal patterns of growth variability and growth-climate relationships

Forest height decreased markedly with increasing altitude (Table 2), while stem density increase and maximum volume decreased but the two correlations were not significant at *p* < 0.05. Only forest height was significantly correlated with the AET gradient across altitudes.

**Table 2:** Correlations of stem density, forest height (H_max_) and maximum volume (V_max_) with altitude and annual evapotranspiration (AET).

	Density (/ha)	H_max_ (m)	V_max_ (m^3^)
Altitude (m)	0.390	−0.687^**^	−0.508′
AET (mm)	−0.364	0.615^*^	0.436

*P* < 0.1, ^*^P < 0.05, ^**^P < 0.01.

In bivariate analysis (Table 3), MS and SD were closely related to forest height with an *R*^2^ of 0.61 and 0.50, respectively, similar to their *R*^2^s with forest type and climate (between 0.36 and 0.62). The six RWI-climate correlations were more closely related with forest type and climate (*R*^2^ between 0.30 and 0.79), but also significantly related with forest height (*R*^2^ between 0.16 and 0.43, except Cor_p6P and cor_p7P). Stem density showed weak bivariate relationships with these RWI-climate correlations (*R*^2^ < 0.25), however, our subsequent multivariate analyses showed that it is also necessary in explaining their altitudinal patterns. Maximum volume, however, was a weaker predictor than forest height for MS and SD, and also for the RWI-climate correlations.

**Table 3:** *R*^2^ for each of altitude, forest type, annual evapotranspiration (AET), stem density, forest height (H_max_) and maximum volume (V_max_) in explaining growth variability and RWI-climate correlations.

Variable	Growth variability		RWI-climate correlations					
	MS	SD	Cor_c6T	Cor_c7T	Cor_c6P	Cor_p7T	Cor_p6P	Cor_p7P
Altitude (m)	+0.500^**^	+0.393^*^	+0.667^***^	+0.652^***^	−0.754^***^	−0.319^*^	+0.432^**^	+0.629^***^
Forest type	0.615^*^	0.578′	0.774^**^	0.804^**^	0.787^**^	0.760^**^	0.662^*^	0.628^*^
AET (mm)	−0.450^**^	−0.358^*^	−0.654^***^	−0.64^***^	+0.764^***^	+0.298^*^	−0.410^*^	−0.662^***^
Density (/ha)	+0.204′	0.136	0.111	0.161	−0.115	−0.249′	0.073	0.001
H_max_ (m)	−0.608^***^	−0.497^**^	−0.425^**^	−0.291^*^	+0.234′	+0.284^*^	−0.191	−0.162
V_max_ (m^3^)	−0.263′	−0.219′	−0.325^*^	−0.172	−0.163	0.028	−0.057	−0.074

MS, mean sensitivity; SD, standard deviation; r2, mean correlation between trees; r3, mean correlation within trees; Cor_c6T, Cor_c7T and Cor_c6P, correlation of RWI with current June and July temperature, and June precipitation, respectively; Cor_p7T, Cor_p6P and Cor_p7P, correlation of RWI with previous July temperature and precipitation, and July precipitation, respectively. +, positive relationships, −, negative ones. *P* < 0.1, ^*^P < 0.05, ^**^P < 0.01, ^***^P < 0.001.

In multivariate analyses (Table 4), forest height is the only predictor retained in the final models (based on BIC model selection) for MS and SD. As for RWI-climate relationships, the four predictors together explained 74% to 97% of variation in the six correlations. Forest type was retained in most models and had the highest importance value (0.45–0.61) in each model. AET was retained in all the models, and its importance (0.13–0.95) was also high in most cases. Despite lower importance (0.05–0.16) than forest type and AET, density and forest height were retained in most models, suggesting that they still played a necessary (though not strong) role in shaping the altitudinal change of RWI-climate correlations. On the other hand, maximum volume was excluded from all the models.

**Table 4:** Importance value (i.e. the proportion that a predictor contributes to the model *R*^2^, see Methods), for each predictor retained in the final model to explain growth variability or RWI-climate correlation.

Variable	Growth variability		RWI-climate correlations					
	MS	SD	Cor_c6T	Cor_c7T	Cor_c6P	Cor_p7T	Cor_p6P	Cor_p7P
Forest type	—	—	**0.46**	**0.55**	**0.45**	**0.61**	**0.60**	—
AET	—	—	**0.33**	**0.36**	**0.41**	0.13	0.23	**0.95**
H_max_	**1.0**	**1.0**	0.16	—	0.09	0.15	0.12	—
Density	—	—	0.05	0.09	0.05	0.11	0.05	0.05
V_max_	—	—	—	—	—	—	—	—
Model *R*^2^	0.61	0.50	0.97	0.96	0.95	0.94	0.82	0.74

The models were obtained through BIC model selection, and “—” denotes dropped terms. In each model the most important one or two terms were boldfaced.

## Discussion

### Altitudinal patterns of growth variability and its drivers

Previous tree-ring studies along geographic gradients generally focus on the change of RWI-climate relationship, instead of the temporal growth variability during climate change. Our study sampled 15 plots across a large altitudinal gradient from temperate to timberline forests. We showed that growth variability (MS and SD) increased at higher altitude on humid mountains, even when different altitudinal zones were included (Fig. 1). Previous studies have noticed that MS and SD changes with altitude. In humid regions MS and SD were generally found to be higher with increasing altitudes^14,16,48^, while the converse altitudinal pattern was commonly observed in arid regions^10,27,49^. This difference is generally explained by the fact that: in arid regions, the water deficit at low altitudes is critical in limiting radial growth, but the stress is alleviated at higher altitudes and thus growth variability is lower^14,27^. Thus, the two contrasting altitudinal patterns are actually consistent, in that MS and SD are higher under harsher climate^28^. In addition to changes along altitude, other studies also found that MS and SD differed markedly among forest types (species) under similar climate (e.g. ref.^21^). For instance, Liang *et al*.^18^ found that MS and SD decrease from early- to late-successional forests in a same site in northeast China. These results suggest that species identity may be another important factor affecting MS and SD in addition to environmental condition.

With these previous results, we had expected that climate and forest types would be the strongest predictors for MS and SD. However, only forest height was retained in the final models (Table 4). This result does not mean that climate and forest type have neglectable influence on MS and SD. Note that AET and forest type significantly explained 36–65% of variance in MS and SD (Table 3), thus they were excluded from the models simply because their collinearity with forest height. Nevertheless, our results do suggest that forest height is a very important predictor for the temporal variability of radial growth.

Forest (tree) height has seldom been used to explain MS and SD before. However, in a few tree-ring studies that have documented forest height (or tree size), we also found evidence that MS (SD) decreased with taller forest height^50^. For instance, in a study that MS and SD increased with higher altitude on a humid mountain, forest height decreased sharply with increasing altitude^14^. Similarly, on an arid mountain in northwest China, MS and SD decreased while tree height increased with higher altitude^27^. In another study along a successional series, MS and SD decreased from early- to late-successional forests, accompanied by an increase in forest height towards late-successional forests^18^. Nevertheless, the potential role of forest height was not explored in these studies. Some studies have examined the influence of DBH, and found that large-DBH trees have lower MS than small-DBH ones^21,51,52^. In all these cases MS (and SD) were negatively related to forest height or tree size, similar to our results (Table 3).

As for why growth variability (MS and SD) is negatively related to forest height, we hypothesis that this is because radial growth will show higher variability when productivity is lower. Forest height is well known to be higher in more productive sites (e.g. ref.^23,25,53,54^), consequently MS and SD are negatively related to tree height (size). Both forest productivity and height decrease with higher altitude on humid mountains but increased on mountains in arid regions. Consequently, MS and SD increased with higher altitude on humid mountains (e.g. this study and ref.^14^) but decreased on arid mountains^27^. This hypothesis may also explain why forest height is a better predictor in our study. Forest productivity is affected by a number of factors in addition to climate, e.g. soil fertility and local topography. Meanwhile, forest height is also affected by both these local factors and climate, and thus may a better indicator of productivity than climate indices. Along successional series, forest productivity generally increases from early- to late-successional stages (e.g. ref.^55^), and forest height also increases towards late-successional forests^18,41^. According to our hypothesis, MS and SD should be lower in later-successional forests, and thus lead to a negative relationship of MS and SD with forest height. This prediction is consistent with the findings in Liang *et al*.^18^. Meanwhile, larger-DBH trees (i.e. taller trees) commonly have higher productivity than smaller-DBH (e.g. ref.^55^). Consequently, smaller-DBH trees are expected to have higher MS, which has been reported in previous studies^21,51,52^. Thus, our hypothesis seems to provide an explanation for different situations (altitudinal gradients in humid and arid regions, successional series and different tree sizes).

However, the above-mentioned hypothesis and still needs further tests. Geographic patterns of MS and SD seem to be related to a number of abiotic and biotic factors, such as temperature gradient, water availability, tree size, age and species identity etc. (e.g. ref.^21,28,56^). Height is a key dimension of tree size, and is closely related to not only DBH and tree age but also productivity. Thus in theory height ought to have a critical in affecting the growth response to climate change. We suggest that more studies are needed to test the relative influence of tree height, age, species identity and climate, for a better understanding of temporal growth variability under climate change.

### Drivers for altitudinal change of growth-climate relationship

Many studies have reported that RWI-climate relationship changes along altitudinal gradients. Summer temperature was generally found to be the limiting climate factor of tree growth at high altitudes, while water-availability was the limiting factor at low elevations^9–11,14,57^. Our results on the altitudinal change of RWI-climate correlations are generally consistent with these previous findings. For instance, RWI was positively related to current June and July temperature in high-elevation plots (Fig. 2), consistent with the “growth limitation hypothesis” that growth of timberline forests is limited by growing season temperature^58,59^. At low altitudes, RWI was positively related to current June precipitation while negatively related to current June temperature, a typical indication that water availability is limiting for radial growth (e.g. ref.^13,57^). This limiting effect disappeared towards high altitude, which is a commonly-reported pattern because of increased precipitation and decreased temperature with higher altitude^9,10,16^. Influence of previous summer climate on current year growth (i.e. the lag effect) is evident in our study. Interestingly, previous and current summer climate showed not only contrast effect on growth but also converse altitudinal pattern (Fig. 2c,d). Similar contrast effects of current and previous summer climate on radial growth have also been found in previous studies in humid regions (e.g. ref.^18,48^), and is generally interpreted by the fact that rapid growth in the current year will reduce the nutrients stored in trees that can be used for next year growth^28,56^.

Our results support the idea that these altitudinal patterns can not be explained by climate gradient alone. Forest type had the highest importance in each model (Table 4). In only one model (Cor_p7P) forest type was excluded, but this is again because the explanatory power of forest type was included in AET and do not mean it has neglectable influence (note that in Table 3 forest type explained 62.8% of variation in Cor-p7P, only slightly lower than the 66.2% of AET). Thus, in contrast to previous studies that have included only one forest zone or species, we conclude that forest type is the most important factor leading to different RWI-climate relationship, while altitudinal climate gradient plays a second role. Recent studies along latitudinal have also suggested that the geographic change of RWI-climate relationship is shaped by climate gradient and forest type together^3,4,9,60^.

Local scale studies have noticed the effects of tree size (age) on RWI-climate relationship. For instance, older trees were found to be more sensitive to climate change than younger ones^22,61,62^, and large-DBH trees were more sensitive to drought than small trees^6,21^. Some authors also showed that the differences in RWI-climate relationships between DBH classes were greater than those between species^51^. These evidences suggest that tree size or height may play a role in affecting RWI-climate relationship. As for the effect of stand density, forest thinning treatments have found that low density stands were less sensitive to droughts than high-density ones, because lower competition increases the resource availability of individual trees^6,63^. Based on these evidences, we had expected that forest height, maximum volume and stem density might play a stronger role in explaining RWI-climate relationship. However, our results showed that the importance of height, volume and density were low (Table 4). Studies that found clear effect of tree size (age) and density were generally conducted by comparing trees of a same species under a similar climate. Our results suggest that height and density are weaker modulators of RWI-climate relationship, when compared with the great gradients of climate and forest type. It should be noted that forest height and density entered most of the final models for the RWI-climate correlations, suggesting they still played a necessary role in addition to forest type and climate. Another possible reason for the weak effect of height and density found here may because our study area is a humid cold region without water deficit^25,33^. Existing evidences on the effect of tree size (age) and density were mostly found for the growth sensitivity to drought^6,21,63^. Thus, we suggest that forest height and density may reveal a much stronger effect in arid regions, which need to be tested carefully in the future.

## Conclusion

In summary, our study showed that both growth variability and RWI-climate relationships varied regularly across different forest types along an altitudinal gradient in northeast China. We also examined the relative importance of climate, forest type, height and density in shaping these altitudinal patterns (probably the first one along altitudinal gradients). We showed that forest height is the most important predictor for MS and SD, while forest type and climate were more important for RWI-climate relationship (with height and density still played a necessary role). The importance of forest type and height highlight the necessity for future studies to test the effect of forest structure (species composition, height and density, etc.), on not only RWI-climate relationship but also growth variability. If these effects are verified across latitudinal (altitudinal) gradients, then forest structure should be considered when predicting global forest growth in response to future climate change.

## Supplementary information

supplementary information
