# Supplementary material for: Forest type and height are important in shaping the altitudinal change of radial growth response to climate change
Source: Sci Rep. 2019 Feb 4;9:1336. doi: 10.1038/s41598-018-37823-w (PMC6362071; doi:10.1038/s41598-018-37823-w)
Supplement: Supplementary file 1 — supplementary information [file 41598_2018_37823_MOESM1_ESM.docx]

**Forest type and height are important in shaping the altitudinal change of radial growth response to climate change**

Penghong Liang, Xiangping Wang, Han Sun, Yanwen Fan, Yulian Wu, Xin Lin and Jinfeng Chang

**Supplementary Table S1** Altitudinal changing rate for mean temperature and total precipitation of each month (from January to December) on Mt. Changbai (Wang et al. 2008).

|  | Jan. | Feb. | Mar. | Apr. | May | Jun. | Jul. | Aug. | Sept. | Oct. | Nov. | Dec. |
| --- | --- | --- | --- | --- | --- | --- | --- | --- | --- | --- | --- | --- |
| Temperature (°C/m) | -0.004 | -0.005 | -0.006 | -0.006 | -0.006 | -0.006 | -0.006 | -0.006 | -0.006 | -0.006 | -0.005 | -0.004 |
| Precipitation (mm/m) | 0.002 | 0.002 | 0.008 | 0.013 | 0.017 | 0.035 | 0.050 | 0.040 | 0.018 | -0.002 | 0.007 | 0.003 |

**Supplementary Table S2** Correlations between ring-width indices and monthly mean temperature or total precipitation.

| Plot | CBS1 | CBS2 | CBS3 | CBS4 | CBS5 | CBS6 | CBS7 | CBS8 | CBS9 | CBS10 | CBS11 | CBS12 | CBS13 | CBS14 | CBS15 |
| --- | --- | --- | --- | --- | --- | --- | --- | --- | --- | --- | --- | --- | --- | --- | --- |
|  | Monthly mean temperature | | |  |  |  |  |  |  |  |  |  |  |  |  |
| p6 | -0.052 | -0.174 | -0.084 | -0.201 | -0.083 | -0.12 | 0.014 | 0.045 | -0.074 | -0.118 | 0.148 | 0.105 | -0.145 | 0.173 | -0.078 |
| p7 | **-0.271 *** | **-0.299 *** | -0.239 ' | **-0.493 ***** | **-0.391 **** | -0.148 | 0.09 | 0.19 | **-0.361 **** | -0.136 | 0.047 | 0.041 | -0.151 | 0.015 | -0.049 |
| p8 | -0.117 | **-0.308 *** | -0.202 | **-0.29 *** | **-0.34 **** | 0.005 | 0.211 | 0.162 | -0.246 ' | -0.06 | 0.032 | -0.03 | -0.036 | -0.004 | -0.113 |
| p9 | **0.333 **** | 0.116 | **0.268 *** | 0.148 | -0.133 | 0.215 ' | -0.082 | -0.161 | 0.168 | -0.213 | 0.089 | -0.169 | -0.078 | 0.007 | -0.115 |
| p10 | -0.005 | -0.099 | 0.028 | 0.104 | -0.026 | 0.203 | 0.014 | -0.104 | 0.023 | -0.171 | **-0.376 **** | 0.06 | -0.075 | 0.07 | -0.055 |
| p11 | -0.146 | -0.207 | -0.184 | -0.062 | -0.008 | -0.109 | 0.071 | -0.05 | -0.094 | -0.132 | 0.005 | -0.227 ' | -0.109 | 0.063 | 0.105 |
| p12 | **-0.28 *** | -0.143 | -0.086 | -0.222 ' | -0.126 | -0.185 | 0.111 | 0.128 | -0.195 | -0.052 | -0.041 | 0.03 | 0.079 | 0.115 | 0.071 |
| c1 | -0.071 | -0.106 | 0.026 | -0.07 | -0.07 | -0.123 | 0.088 | 0.048 | 0.032 | -0.052 | **-0.277 *** | -0.157 | -0.063 | -0.077 | -0.117 |
| c2 | -0.141 | -0.049 | -0.001 | -0.019 | 0.041 | -0.179 | -0.096 | -0.034 | -0.016 | -0.077 | -0.032 | 0.108 | 0.054 | 0.016 | 0.168 |
| c3 | **-0.275 *** | -0.069 | -0.135 | -0.061 | 0.139 | -0.204 | 0.031 | 0.028 | -0.058 | 0.103 | 0.072 | 0.002 | 0.065 | 0.09 | 0.208 |
| c4 | **-0.273 *** | -0.047 | -0.064 | -0.074 | -0.048 | 0.163 | 0.076 | 0.077 | -0.129 | 0.235 ' | 0.244 ' | 0.114 | 0.039 | 0.11 | 0.121 |
| c5 | -0.043 | -0.082 | -0.202 | -0.134 | 0.109 | 0.057 | 0.208 | **0.281 *** | -0.108 | 0.194 | **0.279 *** | -0.107 | -0.129 | -0.052 | 0.117 |
| c6 | **0.291 *** | **0.292 *** | **0.339 **** | **0.287 *** | 0.21 | 0.045 | 0.078 | 0.061 | 0.23 ' | -0.134 | 0.169 | -0.206 | -0.245 ' | -0.22 ' | 0.033 |
| c7 | 0.191 | **0.294 *** | **0.348 **** | **0.313 *** | **0.275 *** | 0.099 | 0.031 | 0.002 | 0.204 | 0.09 | 0.12 | -0.03 | -0.143 | -0.24 ' | 0.038 |
| c8 | 0.191 | 0.11 | **0.256 *** | 0.215 ' | 0.141 | 0.052 | 0.127 | 0.107 | 0.174 | 0.019 | 0.128 | -0.088 | 0.041 | -0.161 | **0.305 *** |
| c9 | -0.026 | -0.003 | 0.019 | 0.028 | 0.094 | -0.027 | 0.005 | -0.068 | 0.008 | -0.123 | **0.331 **** | -0.059 | -0.01 | 0.105 | **0.265 *** |
|  | Monthly total precipitation | | |  |  |  |  |  |  |  |  |  |  |  |  |
| p6 | 0.153 | **0.392 **** | **0.298 *** | 0.227 ' | 0.075 | 0.213 | -0.105 | 0.042 | 0.173 | 0.207 | 0.046 | 0.11 | 0.137 | -0.08 | **-0.265 *** |
| p7 | 0.158 | 0.194 | **0.329 *** | **0.273 *** | 0.007 | 0.049 | 0.131 | 0.174 | **0.279 *** | 0.018 | 0.03 | 0.011 | **-0.319 *** | -0.176 | **-0.313 *** |
| p8 | 0.028 | 0.069 | -0.05 | 0.125 | 0.178 | 0.131 | 0.241 ' | 0.215 ' | -0.019 | 0.062 | -0.069 | -0.014 | -0.036 | -0.114 | -0.022 |
| p9 | -0.013 | 0.156 | 0.134 | 0.115 | 0.077 | 0.2 | **0.262 *** | 0.144 | 0.063 | 0.251 ' | 0.09 | 0.038 | -0.015 | 0.027 | -0.097 |
| p10 | 0.022 | -0.062 | 0.034 | -0.004 | 0.156 | -0.022 | -0.046 | -0.092 | 0.061 | 0.208 | 0.047 | 0.12 | 0.096 | -0.075 | 0.174 |
| p11 | 0.121 | 0.177 | 0.136 | 0.096 | 0.228 ' | **0.286 *** | -0.074 | -0.139 | 0.107 | 0.059 | 0.157 | 0.139 | 0.139 | 0.181 | 0.234 ' |
| p12 | -0.091 | -0.141 | -0.026 | -0.06 | -0.071 | 0.06 | 0.149 | 0.028 | -0.178 | -0.214 | 0.179 | 0.117 | 0.022 | 0.239 ' | 0.193 |
| c1 | 0.074 | 0.028 | -0.018 | 0.085 | -0.023 | 0.23 ' | 0.035 | -0.018 | 0.051 | -0.054 | 0.018 | -0.017 | 0.077 | -0.006 | -0.011 |
| c2 | -0.109 | -0.134 | -0.116 | -0.055 | -0.032 | -0.078 | 0.112 | -0.009 | 0.02 | -0.01 | -0.127 | 0.002 | 0.05 | -0.034 | 0.151 |
| c3 | -0.09 | **-0.271 *** | **-0.281 *** | -0.035 | 0.057 | -0.044 | -0.173 | -0.157 | -0.137 | -0.13 | -0.088 | -0.133 | 0.096 | 0.066 | 0.138 |
| c4 | 0.129 | 0.02 | 0.043 | -0.1 | 0.012 | -0.011 | -0.027 | 0.044 | 0.062 | 0.102 | 0.077 | -0.228 ' | 0.017 | -0.095 | 0.093 |
| c5 | 0.087 | 0.152 | 0.22 ' | 0.217 ' | 0.206 | -0.081 | -0.065 | -0.118 | 0.235 ' | -0.094 | -0.126 | 0.129 | -0.062 | -0.041 | -0.06 |
| c6 | -0.158 | **-0.285 *** | -0.228 ' | -0.198 | -0.142 | -0.059 | 0.016 | -0.039 | -0.145 | -0.033 | -0.091 | 0.108 | **0.299 *** | **0.373 **** | 0.219 ' |
| c7 | -0.024 | -0.142 | -0.09 | -0.031 | -0.104 | 0.046 | 0.017 | -0.006 | -0.076 | -0.062 | -0.049 | -0.023 | -0.023 | -0.14 | 0.117 |
| c8 | 0.169 | 0.184 | 0.049 | -0.082 | -0.132 | 0.15 | 0.065 | 0.125 | 0.023 | -0.083 | -0.085 | 0.013 | 0.044 | -0.044 | -0.151 |
| c9 | -0.054 | 0.023 | 0.062 | -0.032 | -0.079 | 0.061 | 0.135 | 0.076 | -0.137 | 0.104 | 0.16 | 0.175 | 0.228 ' | **0.295 *** | 0.011 |

Abbreviations: p6~p12, climatic indices from June to December of the previous year; c1~c9, climatic indices from January to September of the current year. ‘*P* < 0.1, **P* < 0.05, ***P* < 0.01, ****P*< 0.001.

**Supplementary Figure S1**

(a) Principal components analysis (PCA) for the correlations between RWI and monthly climate variables of the 15 chronologies (see Supplementary Table S2). (b) PCA1 scores for the correlation of RWI with monthly temperature. (c) PCA1 scores for the correlation of RWI with monthly precipitation. In (b) and (c), filled columns denotes the months with the highest PCA1 scores; p6~p12, June to December of the previous year; c1~c9, January to September of the current year.
